# How does the implementation of AI-based automation of administrative tasks affect healthcare professionals’ work? Study protocol for a longitudinal embedded case study in Swedish primary and specialist care

**DOI:** 10.1136/bmjopen-2025-110553

**Published:** 2026-03-26

**Authors:** Luís Irgang, Petra Svedberg, Jens Nygren, Lena Petersson

**Affiliations:** 1 School of Health and Welfare, Halmstad University, Halmstad, Sweden

**Keywords:** Artificial Intelligence, Digital Technology, Health Workforce, Implementation Science, Primary Care, Administration

## Abstract

**Abstract:**

**Introduction:**

Artificial intelligence (AI) is transforming healthcare through enhanced computational capabilities that process vast amounts of data with unprecedented speed and precision. AI-powered administrative systems hold significant promise for reducing the substantial documentation burden on healthcare professionals, potentially improving operational efficiency and job satisfaction. However, critical knowledge gaps exist regarding how AI implementation affects professional boundaries, work identities and the healthcare work environment. This study protocol describes the Project EFAAI (Efficient and Flexible Working Life in Healthcare with the Support of Digital Tools and AI), which aims to explore how the implementation of AI-based automation of administrative tasks affects healthcare professionals’ work.

**Methods and analysis:**

This research employs a single-embedded case study with a longitudinal approach, following the implementation of an AI-powered healthcare administration system across 12 primary care facilities and 20 specialist care facilities of a major private healthcare provider in Sweden. Data collection spans 18 months through three sequential waves at 6-month intervals. The project consists of three work packages (WP): WP1 explores changes in professional boundaries and AI literacy requirements using exploratory meetings with healthcare leaders and in-depth semistructured interviews with 20 physicians from primary care and 20 from specialist care (n=40); WP2 investigates implementation barriers and facilitators through in-depth semistructured interviews with the same 40 physicians as WP1 for longitudinal follow-up; and WP3 examines the impact on efficient and flexible work conditions using a mixed-methods approach integrating quantitative operational and administrative data from the healthcare provider’s information systems and administrative databases (eg, documentation completion times, consultation duration, workload distribution metrics, operational efficiency ratios) with qualitative data from four focus groups and workshops with physicians, nurses, healthcare leaders and administrative staff.

**Ethics and dissemination:**

The study adheres to the ethical guidelines of the Swedish Research Council and complies with the regulations of the Swedish Ethical Review Authority. Data will be collected with informed consent from all participants, anonymised and stored in accordance with General Data Protection Regulation (GDPR) requirements. Results will be disseminated through international publications, conferences, teaching activities and stakeholder engagement.

**STRENGTHS AND LIMITATIONS OF THIS STUDY**

- The longitudinal embedded case study design with 18-month follow-up allows for a comprehensive evaluation of implementation processes and temporal changes in professional experiences and system integration over time.
- The study uses a mixed-methods approach that includes semistructured interviews, focus groups, workshops and quantitative operational metrics, allowing methodological triangulation and a comprehensive understanding of artificial intelligence (AI) implementation impact.
- Real-world implementation setting in an active healthcare environment enhances validity and practical applicability of findings to clinical practice.
- The study setting within a major European healthcare provider offers access to diverse professional contexts across primary and specialist care, though the focus on physicians excludes perspectives of nurses, administrative staff and other healthcare professionals who interact with the AI system daily.
- The study begins after the AI system implementation has commenced, potentially missing critical preimplementation data on foundational decisions, expectation setting and organisational readiness activities that significantly influence implementation trajectories.

## Introduction

 Artificial intelligence (AI) is revolutionising healthcare and is positioned to address numerous pressing challenges within the sector.^1 2^ AI introduces computational capabilities that can process vast amounts of data with unprecedented speed and precision, potentially transforming how healthcare is delivered, managed and experienced.^3^ Current applications span from robot-assisted surgery, where systems like Da Vinci provide enhanced precision and control,^4^ to deep learning algorithms that analyse medical images with accuracy comparable to or exceeding human specialists.^5^ These AI systems promise improved health outcomes, reduced per capita costs, enhanced working conditions for healthcare professionals, increased accessibility and better patient care experiences.^6^,^9^

Among these applications, AI-powered administration systems for automating non-clinical tasks have gained significant prominence. These systems integrate natural language processing, machine learning algorithms and workflow automation to streamline healthcare administrative processes.^10 11^ By enhancing information flow between departments, reducing documentation errors, facilitating faster information retrieval and creating more transparent coordination of care, these systems address a critical need in healthcare delivery.^12 13^ Their primary value lies in reducing the substantial administrative burden that healthcare professionals encounter daily, including documentation in electronic health records, medical coding, appointment scheduling and managing patient correspondence.^14 15^ Research has demonstrated tangible benefits, with ambient AI systems using voice recognition reducing documentation time varying from 26% to 40%.^16 17^ This efficiency gain releases healthcare staff to engage in activities requiring human expertise and personalised care,^18^ which may result in improved operational efficiency, enhanced job satisfaction, reduced burnout rates and improved patient satisfaction.^19 20^

While the implementation of AI in healthcare can contribute to increased quality, efficiency and improved working environments,^21^ realising these benefits requires careful consideration of how AI technologies reshape professional roles, relationships and work processes. This is particularly critical as multiple tensions may arise during implementation, including power fluctuations, challenges to professional identity, the dilemma of balancing new technological knowledge with traditional medical expertise and increased performance pressures from managers, patients and other stakeholders.^22^ Despite growing research interest in AI implementation and its implications for healthcare workers, several critical knowledge gaps remain unaddressed.

First, there is a pressing need to understand healthcare professionals’ responses toward AI implementation in healthcare settings, particularly regarding professional boundaries and work identities. Workplace changes typically generate ambivalence, manifesting as concerns about impacts on professional identity, responsibilities and overall work situations.^23^,^25^ AI implementation represents a particularly profound change as it potentially redefines core aspects of professional practice, including decision-making authority, task allocation and interprofessional relationships.^1^ Knowledge of healthcare professionals’ responses to AI-driven changes is therefore critical, as these responses significantly influence adoption patterns, implementation success and ultimately, the realisation of AI’s potential benefits.^26^

Second, despite extensive discourse and expectations regarding AI’s potential positive impact on healthcare professionals, empirical evidence validating these expectations remains limited. Research demonstrates that healthcare professionals’ acceptance of AI is conditioned by their perception of its potential to enhance their performance.^27^ However, there is a lack of empirical evidence on how AI automation of healthcare routines affects health outcomes in real-world settings.^28^ Healthcare professionals may experience varied outcomes, from enhanced autonomy and job satisfaction to deskilling, reduced professional discretion or increased standardisation.^29^ These diverse experiences highlight the complexity of value creation through AI implementation and underscore the need for nuanced, context-sensitive research examining both intended and unintended consequences for healthcare professionals’ work experiences and professional identities.^30^

Third, research on AI in healthcare has been constrained by a narrow technology-centric perspective that prioritises technical design over understanding the complex socio-technical healthcare ecosystem.^31^ Significant challenges in AI adoption lie not in the technology itself but in its implementation and integration into daily clinical practice.^32^,^34^ This ‘last mile’ implementation challenge represents a critical barrier to realising AI’s potential benefits and calls for implementation science approaches that bridge the gap between technological capabilities and practical clinical utility.^35 36^ This research gap necessitates a more comprehensive understanding of implementation barriers and facilitators from healthcare professionals’ perspectives.^37 38^ Their firsthand experiences with implementation challenges may provide invaluable insights that could inform the development of more effective, context-sensitive implementation strategies and guide the adaptation of implementation approaches for different AI applications in varied healthcare settings.

Fourth, while it is well established that the successful implementation of emerging technologies like AI requires appropriate technological knowledge—AI literacy—prior literature lacks comprehensive explanations of this concept. AI literacy refers to the understanding, capability and skills in using AI technologies.^39^ Healthcare professionals and organisations need to develop AI literacy to improve adoption and usage of AI applications.^40^ However, a significant gap remains in understanding what constitutes relevant AI literacy for healthcare professionals implementing and using AI in their routines. Specifically, there is limited understanding regarding the kind, level, content and depth of knowledge healthcare workers need about AI systems to trust and properly use them in daily routines.^41^ This knowledge gap impedes the development of effective training programmes and educational interventions that could support successful AI implementation in healthcare settings.

Against this backdrop, this project aims to explore how the implementation of AI-based automation of administrative tasks affects healthcare professionals’ work. The purpose of this study protocol is to describe the main foundations, rationale, design and methodology of Project EFAAI—Efficient and Flexible Working Life in Healthcare with the Support of Digital Tools and AI.

### Research objectives

The specific research objectives (RO) of Project EFAAI are:

RO1—to explore how boundaries around the healthcare professionals’ work change when an AI-powered healthcare administration system is implemented at their workplace.

RO2—to characterise what is relevant to AI literacy for healthcare professionals in an AI-powered healthcare ecosystem.

RO3—to explore barriers and enabling factors in the process of implementing AI-powered healthcare administration systems and to understand how the technology becomes or does not become part of everyday routines (embedded) and is transformed into sustainable practice (integrated).

RO4—to analyse how and to what extent AI automation of administrative work in healthcare can enhance efficient and flexible work conditions and provide value for healthcare professionals.

## Methods and analysis

### Study design

The Project EFAAI draws on a single-embedded case study with a longitudinal approach,^42^ focusing on the implementation of an AI-powered healthcare administration system within a private healthcare provider. Embedded case studies facilitate the systematic interpretation, organisation and integration of diverse knowledge sets emerging from complex cases through the analysis of distinct subunits.^43^ This approach is particularly advantageous for exploring contextual factors that influence implementation processes and outcomes across multiple settings,^44^ while also enabling investigation of individual attitudes and behaviours toward technological innovations across different organisational units.^45^ The longitudinal dimension enables identification of critical implementation events^42^ and has demonstrated significant value in implementation research by facilitating the exploration of temporal changes in implementation conditions and underlying processes.^46^

To gain a nuanced understanding of the evolving dynamics of the research phenomenon, we will conduct serial data collection over three time intervals: 6 months, 12 months and 18 months. This sequential approach enables documentation of evolving perspectives, experiences and insights over time.^47^

### Study setting

This project is conducted in co-production with the Innovation & Partnership Hub at Capio Ramsay Santé^48^ (https://www.ramsaysante.eu/). Ramsay Santé, a leading private hospital and primary care provider in Europe, employs 36 000 staff and 8600 practitioners, catering to 7 million patients across 350 facilities in France, Sweden, Norway, Denmark and Italy. Since 2018, Capio, a Swedish healthcare provider, has been part of Ramsay Santé, contributing approximately 12 000 employees in Sweden. The study setting will focus on 12 primary care facilities and 20 specialist care facilities at Capio in Sweden that have implemented the AI system. Primary care serves as the first point of contact in the healthcare system and is responsible for managing a broad range of health conditions across a large patient population. In contrast, specialist care units provide services to patients referred for more targeted or advanced medical evaluation and treatment. These settings represent different aspects of the healthcare sector, each presenting varying complexity in terms of administrative tasks, including the amount of documentation in medical records, the structure of documentation, the nature of the meetings with patients, the workforce management and time burden, as well as patient trajectory.

### The AI system

The AI-powered healthcare administration system under implementation at Capio Ramsay Santé is a commercial platform developed by a Swedish health-tech company certified under the European Union Medical Device Regulation (MDR) as a Class IIa medical device.^49^ The platform was launched in 2023 and has been tested and implemented across multiple specialties in primary, secondary and hospital care settings in Sweden.

The AI system aims to streamline administrative tasks, reducing the time burden on healthcare professionals and improving patients’ care trajectories. The technological architecture centres on ambient scribe technology, which uses voice activation to capture physician-patient conversations during clinical encounters without requiring active dictation or typing. Through advanced natural language processing (NLP) and speech recognition algorithms, the ambient scribe listens to natural dialogue between clinician and patient, automatically identifies clinically relevant information and generates structured clinical notes in real-time. These conversational data are then processed by machine learning algorithms that analyse the consultation content and suggest appropriate diagnostic codes and procedure codes, thereby reducing manual coding time while improving coding accuracy. The system further employs large language models to transform this captured content into concise visit summaries, referral letters and patient communication materials, creating structured, professional documentation from conversational exchanges. The platform also incorporates enhanced coordination tools and information-sharing capabilities that facilitate communication among physicians, nurses, psychologists, occupational therapists and secretaries.

The system operates across three key phases of clinical workflow. In the preparation phase—before meeting with patients—it provides technical support for previsit planning and documentation organisation. During the consultation phase, the ambient scribe facilitates real-time documentation through speech-to-text capabilities and automated coding, enabling healthcare professionals to maintain focus on patient interaction rather than manual data entry. In the post-consultation phase, the system supports documentation completion and ensures transparency of care activities for both clinical staff and patients through its information-sharing platform.

This technological integration presents new opportunities and challenges for operational practices. Healthcare leadership at Capio foresees several difficulties in redesigning workflows and responsibilities, which require thorough exploration, making this an ideal setting for implementation research.

### Participant recruitment and sampling strategy

Physicians are the primary focus for addressing RO1, RO2 and RO3, as they bear primary responsibility for the clinical documentation tasks most directly affected by the AI system and are the professional group most consequentially exposed to boundary negotiations when AI assumes parts of their administrative work.^22 50^ Nurses, administrative staff and healthcare leaders are additionally included to address RO4 to broaden the analytical scope across stakeholder groups.

To select and recruit potential participants, site leaders and secretaries will provide a list of healthcare professionals actively using the AI system at each selected site, stratified by care setting (primary and specialist). From this list, we will use purposive sampling^51^ to recruit participants with different genders, working experience and diverse clinical specialties (for specialist care, representing different specialisation areas such as internal medicine, psychiatry and orthopaedics), but with similar time of exposure to the AI-powered healthcare system. This sampling strategy ensures diversity in demographic and professional characteristics while controlling for implementation experience, allowing us to capture varied perspectives on professional boundary work and AI literacy requirements among participants at comparable stages of AI system implementation.

Potential participants will be informed by the secretary and site leadership about the research. The authors of the study will schedule interviews, focus groups and workshops with the support of the secretaries, ensuring voluntary participation. Participants will receive written information about the study, including its aims, time commitment expectations, confidentiality protections and the right to withdraw at any time. If participants withdraw during the study period, we will recruit replacement physicians using the same purposive sampling approach to maintain sample diversity and size.

**Figure 1:**
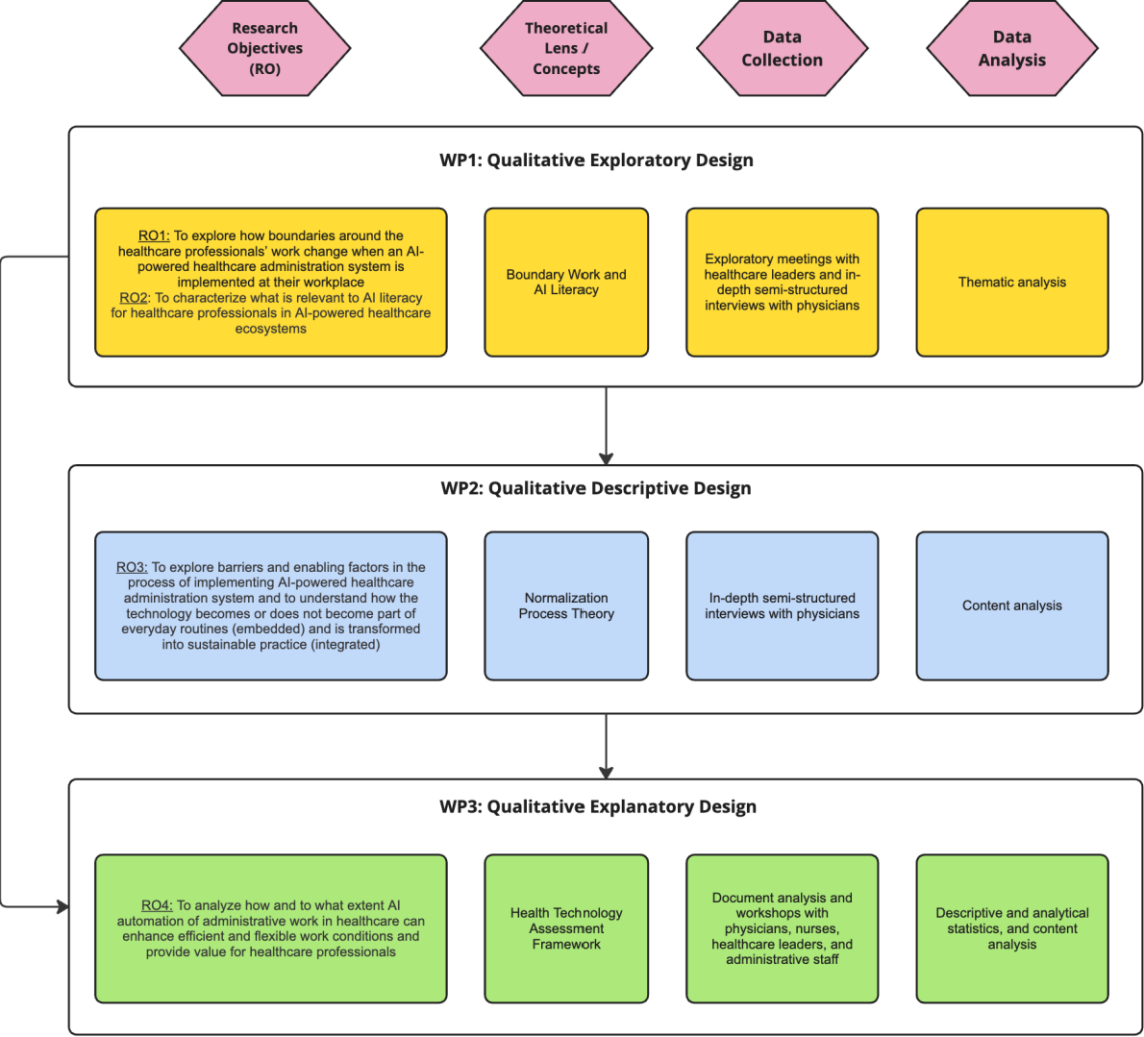
Overview of the research methods and designs of the three work packages (WPs). AI, artificial intelligence; RO, research objective.

This project is structured into three work packages (WP) aligned with the research objectives (figure 1). To ensure methodological fit,^52^ we will employ a mixed-method approach of exploratory, descriptive and explanatory sequential designs, integrating qualitative and quantitative data collection and analysis techniques.^53^ WP1 adopts an exploratory approach, appropriate for investigating underexplored phenomena.^53^ This methodological choice reflects the limited existing research on real-world implementation of AI applications for healthcare administrative automation.^54^ WP2 follows a qualitative descriptive approach,^55^ aiming to provide detailed descriptions of the implementation process based on individual experiences while producing research findings with a high level of adherence to the empirical data. This approach has proven particularly useful for identifying and describing individuals’ activities and interactions that facilitate or hinder the implementation of changes within healthcare settings.^56^ WP3 takes an explanatory approach to provide a detailed understanding of the efficient and flexible work conditions for healthcare professionals facilitated by an AI-powered healthcare administration system. This involves examining quantitative data, complemented by qualitative data to contextualise and enhance the findings.^57^

### WP1: Exploring boundaries and literacy

WP1 will be carried out during the initial 9-month period following the implementation of the AI-powered healthcare administration system, spanning June 2025 to February 2026. In this WP, we will employ an exploratory qualitative design with an abductive approach^58^ to explore two primary aspects: the transformation of healthcare professionals’ work boundaries following the AI system implementation (RO1) and the identification of what is relevant to AI literacy for healthcare professionals operating within an AI-powered ecosystem (RO2).

The data collection in WP1 encompasses two sequential phases of exploratory meetings and in-depth semistructured interviews with healthcare professionals actively engaging with the AI application across the two study units. The initial phase consists of exploratory meetings with healthcare leaders to establish a comprehensive understanding of the implementation process and organisational structure. These preliminary meetings serve to refine our research objectives and enhance our understanding of the empirical phenomenon.^59^ The second phase involves in-depth semistructured interviews with 20 physicians from primary care and 20 physicians from specialist care, guided by a thematic interview guide rooted in the concepts of professional boundary work and AI literacy. These interviews aim to explore the physicians’ perspectives regarding professional boundaries, boundary work, AI application materiality and AI literacy requirements. The interviews are conducted digitally, audio-recorded and last approximately 30–45 min. Data collection in this WP began in June 2025 and is expected to end by April 2026.

To analyse the data, we will employ a thematic analysis technique following a hybrid approach that combines inductive and deductive coding and theme development.^60^ The analysis begins with comprehensive readings of interview transcripts, followed by systematic identification and inductive coding of relevant text and quotations^58^ aligned with the study objectives. During this initial phase, each transcribed interview undergoes multiple readings to ensure thorough comprehension, followed by a systematic coding process to identify patterns, themes and significant statements within the data. Subsequently, a deductive analysis phase commences, wherein inductively derived codes are systematically organised into predefined themes and subcategories established in the theoretical framework. This analytical process employs an iterative approach, continuously moving between empirical data, theoretical constructs and emerging explanations to ensure robust and theoretically grounded findings.^58^

### WP2: Exploring implementation challenges

WP2 will be carried out 6 months after the first wave of interviews. In this WP, we aim at investigating critical elements for the sustainable implementation of AI in healthcare. The primary objective is to investigate both barriers and facilitating factors in the implementation process, with particular focus on how the AI-powered healthcare administration system becomes integrated into daily routines (embedded) and evolves into sustainable practice (integrated) (RO3). Normalisation Process Theory (NPT) was selected as the analytical framework for this work package because it specifically addresses how complex interventions become or do not become part of everyday routines (embedded) and are transformed into sustainable practices.^61^

Data collection will be based on in-depth semistructured interviews with the same participants from WP1 (ie, 20 physicians from primary care and 20 physicians), thus maintaining consistent participant engagement throughout the study period.^47^ The interview guide follows a thematic structure firmly grounded in the four fundamental dimensions of NPT: sense-making, engagement, collective action and evaluation/monitoring.^61^ Based on a qualitative descriptive approach,^55^ we aim to obtain rich narratives of stakeholder experiences and perceptions regarding the AI-powered system implementation. Interviews will be conducted through digital platforms, audio-recorded, with each session designed to span approximately 45 min.

The analysis of data will be systematically conducted using the NPT framework, applying a qualitative content analysis technique^62^ anchored in the generative mechanisms outlined in NPT.^61^ All transcribed interviews will be read several times and deductively coded by two researchers independently. The coded data will be organised into the preliminary codes and potential subcategories. The analytical process will be discussed continuously within the research team to enhance the trustworthiness and rigour of the analysis.

### WP3: Exploring impact

WP3 will be carried out 18 months after the AI-powered healthcare administration system has been implemented. This WP employs an explanatory mixed-methods design^53^ to examine how the implementation of an AI-powered healthcare administration system enhances efficient and flexible work conditions for healthcare professionals (RO4). Drawing from the Health Technology Assessment (HTA) framework,^63^ we will integrate quantitative operational and administrative data to evaluate the efficiency of the healthcare administration system and qualitative experiential data to develop a comprehensive understanding of the system’s real-world impact within the healthcare context.

The data collection in WP3 will follow three overlapping stages:

Stage 1. The initial phase focuses on gathering and analysing secondary quantitative data from the healthcare provider’s information systems and administrative databases, including operational metrics such as patient appointment scheduling efficiency and patient waiting times, documentation completion times, consultation duration and administrative task processing speeds. Human resource data will encompass staff time allocation patterns and workload distribution metrics. Financial indicators will include administrative costs per patient encounter, operational efficiency ratios and cost-benefit analyses related to administrative processes. This comprehensive dataset will enable the assessment of the administration system’s efficacy and identification of optimal operational processes. This evaluation will serve as a knowledge base to be used on stages 2 and 3.

Stage 2. We will conduct focus groups with various professional groups (eg, physicians, registered nurses, medical secretaries, healthcare managers) across both business areas to gather qualitative data about their experiences, perceptions and perspectives regarding the value and effectiveness of the administration system. Focus groups are a dynamic and interactive means of eliciting in-depth insights from participants, fostering rich discussions and uncovering diverse perspectives within a group setting.^64^ Four focus groups will be conducted (two in primary care and two in specialised care), structured around findings from stage 1. Participant selection will follow purposive sampling to ensure representation of diverse professional backgrounds and experiences.^51^ All sessions will be audio-recorded and transcribed verbatim to maintain data integrity.

Stage 3. We will conduct a series of workshops with physicians, nurses, healthcare leaders and administrative staff from the two business areas of the healthcare provider. These workshops will serve dual purposes: facilitating the integration and comparative analysis of quantitative and qualitative findings, while functioning as a triangulation mechanism to enhance research validity.^65^ Workshop participants will engage in structured discussions to validate and refine emerging themes and patterns, contributing to a holistic understanding of the AI system’s role within the healthcare ecosystem. For instance, while WP1 and WP2 focused specifically on physicians’ experiences with AI implementation, here, we expand the participant scope to include other professional groups whose work is directly affected by the administrative AI system, aiming at gathering a more nuanced understanding of the AI system’s impact across different stakeholder groups.

In WP3, the quantitative data will be analysed through descriptive statistics and linear regression analysis to examine the causal impact of AI system implementation on organisational performance measures. The descriptive analysis will provide baseline measurements and characterise the distribution of key performance indicators, while linear regression models will be employed to quantify the relationship between AI implementation (as the independent variable) and outcome measures, including documentation time reduction, administrative task completion rates and staff workload distribution (as dependent variables).

Focus groups’ data will be analysed through qualitative content analysis^62^ using the HTA framework to examine relationships between implemented technology, observed impacts and contributory mechanisms. The qualitative analysis will specifically focus on explaining why certain quantitative outcomes occurred, how healthcare professionals experience the measured changes, and what factors facilitate or hinder the achievement of efficiency and flexibility goals. For instance, if quantitative data show reduced documentation time, qualitative analysis will explore healthcare professionals’ perceptions of this change, identifying whether time savings translate into meaningful improvements in work conditions. Two researchers will independently conduct deductive coding of transcribed data, followed by systematic organisation into preliminary codes and subcategories.

Workshop sessions will facilitate the synthesis of both qualitative and quantitative data, enabling the development of explanatory models based on the intersection of measurable outcomes with experiential factors, ultimately providing a holistic understanding of how AI automation creates value for healthcare professionals. The analytical process will be discussed continuously within the research team to enhance methodological rigour and trustworthiness.^66^

### Patient and public involvement

None.

## Ethics and dissemination

The study adheres to the Declaration of Helsinki^67^ and the guidelines on research ethics issued by the Swedish Research Council.^68^ As no sensitive personal information will be collected, formal ethical approval from the Swedish Ethical Review Authority is not required under Swedish regulations. Formal ethical review by the Swedish Ethical Review Authority (Etikprövningsmyndigheten (Swedish Ethical Review Authority (*Etikprövningsmyndigheten*): https://etikprovningsmyndigheten.se/en/aboutthe-authority/. Contact email address: registrator@etikprovning.se)) is not required under the Swedish Ethical Review Act (SFS 2003:460) because the study fulfils none of the four conditions that require mandatory review: (1) it involves no physical intervention on living or deceased persons; (2) it uses no method aimed at affecting participants physically or mentally, nor does it involve an obvious risk of harm; (3) it does not involve biological material traceable to a person; and (4) it does not process sensitive personal data as defined under GDPR Article 9 (eg, health data, ethnic origin, political opinions) or personal data relating to criminal offences.^69^ Participants are interviewed exclusively about their professional experiences and workplace practices.

The study upholds the principles of informed consent, respect for privacy and is guided by the ethical principles of autonomy, beneficence, non-maleficence and justice.^70^ Potential participants will receive information about the study and its purpose in a prenotification email and a cover letter, stating that participation is voluntary, that withdrawal at any time without explanation is permitted, and that the confidentiality of the treatment and presentation of data will be maintained. Informed consent will be obtained from all study participants. To ensure confidentiality, anonymisation will be applied across findings to eliminate identifying information, with pseudonymisation employed for direct quotations using generic identifiers. All personal data will be registered according to the General Data Protection Regulation (GDPR 2016/679) and stored in accordance with the Archive Act in the Swedish Code of Statutes (SFS 1990:782).

The research outcomes will be communicated and used in several ways to influence research, policy and practice around the implementation of AI systems in healthcare. Results will be communicated through four planned publications in international peer-reviewed journals and presented at scientific conferences. Results will also be disseminated through teaching activities in higher education programmes, including nursing and health education, and master’s and doctoral courses for various care professions at the hosting university and the National Research School in Health Innovation.

Research findings will also be shared through established networks with private and regional healthcare providers, and with AI Sweden, the Swedish national centre for applied AI, which has over 120 partners across the private, public and academic sectors. A dedicated project webpage will be created to reach healthcare representatives and the wider public. The project will also seek to engage key stakeholders, including patient organisations, professional associations and policymakers, to support the uptake of the findings into practice and policy.

## Discussion

The EFAAI project addresses critical knowledge gaps concerning the implementation and impact of AI-powered healthcare administration systems in real-world clinical settings. By exploring how the implementation of AI-based automation of administrative tasks affects healthcare professionals’ work, the project outcomes will offer theoretical, practical and managerial contributions.

### Theoretical contributions

The EFAAI project makes four theoretical contributions. First, the study expands knowledge about professional boundary work in technology-enhanced healthcare environments by examining how boundaries between healthcare professionals and AI systems evolve over time. This investigation contributes to theoretical frameworks concerning professional identity and role transformation in increasingly automated workplaces,^25 71^ addressing a critical knowledge gap as healthcare undergoes digital transformation.

Second, the project contributes to current debates on task automation in professional services^72 73^ by examining health outcomes derived from the implementation of an AI-powered healthcare administration system. While previous studies suggest that AI systems and tools can automate administrative tasks and potentially redirect clinical time to patient care,^16^ the EFAAI project will extend this understanding by examining the multidimensional impact of such automation. Additionally, the project contributes to contemporary discussions about value creation in healthcare^30^ by examining how AI-powered systems generate different forms of value for various stakeholders within the healthcare ecosystem. The project’s longitudinal approach will allow the development of process models explaining how value emerges, evolves and becomes institutionalised throughout the implementation journey.

Third, this project will contribute to implementation science literature^61 74^ by providing empirical evidence on the implementation of AI-powered healthcare administration systems. By examining how AI systems become normalised (or fail to normalise) within healthcare routines, the project will refine a theoretical understanding of technological embedding processes specific to AI applications.^54^ Furthermore, the project’s longitudinal approach enables theoretical insights into the temporal dimensions of AI implementation that remain underdeveloped in current research.^75^

Fourth, this project will advance a theoretical understanding of AI literacy and its role in the implementation process.^39^ By examining healthcare professionals’ experiences and perspectives during actual implementation, we intend to develop a contextually grounded conceptualisation of AI literacy in healthcare. This research will address essential dimensions of AI knowledge—technical understanding of algorithmic processes, clinical integration knowledge and ethical considerations—required for effective implementation and utilisation.

### Practical and managerial contributions

The findings from the EFAAI project will have practical and managerial implications for healthcare organisations seeking to implement AI-powered administrative systems. Healthcare professionals spend up to 50% of their workday on administrative tasks rather than direct patient care,^76^ representing a substantial opportunity for AI-driven optimisation. By examining the implementation of administrative automation in real-world clinical settings, this project will provide evidence-based guidance that healthcare organisations can use as a basis for their AI implementation strategies and decisions.

The evaluation of the impacts of AI implementation on healthcare professionals’ work conditions and efficiency will provide insights into the actual value created by AI technology, moving beyond theoretical promises to document specific benefits and potential drawbacks. These findings will enable healthcare organisations to make informed decisions about resource allocation, workforce planning and workflow redesign, and professional development when implementing similar systems.

Last, the project’s exploration of boundary work will help healthcare organisations develop appropriate change management approaches. Understanding how professional roles evolve following AI implementation will enable leaders to proactively address potential sources of resistance and support healthcare professionals through the transition process. This knowledge has direct implications for training programmes, workflow redesign initiatives and organisational restructuring efforts that may accompany AI implementation. Furthermore, the clarification of relevant AI literacy for healthcare professionals may have implications for the development of educational interventions and training programmes. This will also help organisations balance the need for adequate AI literacy against the risk of overwhelming staff with excessive technical training.

### Limitations

This project has methodological limitations that should be acknowledged. First, the embedded case study approach, while offering depth and contextual understanding, restricts the generalisability of findings to other healthcare settings. Although this design enables comprehensive exploration of implementation processes within the selected units, the findings may not be fully transferable to healthcare organisations operating under different regulatory frameworks, organisational cultures or resource constraints. Moreover, the project’s focus on two specific business areas within a single healthcare provider limits the exploration of challenges related to the AI system interoperability with other organisations within the healthcare system.

Second, the sample size and composition have specific limitations. The study includes interviews with 20 physicians from primary care and 20 physicians from specialist care, which may not fully represent the diverse perspectives within the broader healthcare system. For instance, WP1 and WP2 focus exclusively on physicians, excluding other healthcare professionals such as nurses and secretaries who may use the AI system daily. This limitation may result in missing insights about how the AI system affects different professional roles and interdisciplinary collaboration. Furthermore, although the longitudinal approach helps address concerns about participant dropout, the potential for participants to leave the study over the 18-month period could affect the consistency and comprehensiveness of the data collected.

Third, the study primarily focuses on stakeholders within the healthcare organisation. Data collection methods will mainly include interviews, workshops and focus groups with nurses, physicians, healthcare leaders and administrative staff, thus not considering other important external stakeholders. For instance, technology providers could provide valuable insights into implementation challenges and system capabilities, as well as patients, whose experiences and perspectives on AI-mediated care could shed light on key aspects of implementation barriers and outcomes.

Lastly, the project’s timing imposes temporal constraints. By focusing on an ongoing implementation process, the study may lack data from critical preimplementation stages where foundational decisions and preparations occur. This could limit an understanding of initial boundary negotiations, expectation setting and organisational readiness activities that significantly influence implementation trajectories.
