# Supplementary material for: How does the implementation of AI-based automation of administrative tasks affect healthcare professionals’ work? Study protocol for a longitudinal embedded case study in Swedish primary and specialist care
Source: BMJ Open. 2026 Mar 26;16(3):e110553. doi: 10.1136/bmjopen-2025-110553 (PMC13034228; doi:10.1136/bmjopen-2025-110553)
Supplement: Supplementary file 1 [file bmjopen-2025-110553.reviewer_comments.pdf]

PEER REVIEW HISTORY

BMJ Open publishes all reviews undertaken for accepted manuscripts. Reviewers are asked to complete a checklist review form and are provided with free text boxes to elaborate on their assessment. These free text comments are reproduced below.

ARTICLE DETAILS

Title (Provisional)

How does the implementation of AI-based automation of administrative tasks affect healthcare professionals’ work? Study protocol for a longitudinal embedded case study in Swedish primary and specialist care

Authors

Irgang, Luís; Svedberg, Petra; Nygren, Jens; Petersson, Lena

VERSION 1 - REVIEW

**Reviewer**	**1**
**Name**	**Bradley, Fay**
**Affiliation**	**The University of Manchester, Division of Nursing, Midwifery & Social Work, School of Health Sciences**
**Date**	**12-Feb-2026**
**COI**	**None**

This protocol outlines a longitudinal case study design comprising three work packages, using mixed methods to explore the implementation of an AI powered healthcare administration system in one private healthcare provider in Sweden. It is well-written and the focus on professional boundaries, AI literacy requirements, implementation challenges and impact on work conditions suggest this will be a very interesting, relevant and timely piece of research.

I have a few suggestions, mainly around clarity/rationale:

1) Setting - it isn't clear whether the sample is from one particular provider site or across several sites. The authors state that the provider (Capio) has 12,000 employees in Sweden. How are the sites to be selected?

2) Sample - linked to this, how will the 20 physicians from PC and 20 physicians from specialist care be identified and approached?

3) the AI system - the detail provided is a bit vague. Is it a bespoke system created by/for Ramsay Sante or is it utilised elsewhere? Has there been any other pilot or evaluation of the system elsewhere?

4) I would move the sections above describing setting, sample and AI system to nearer the start of the methods, after the 'overall design' section. Readers need this context and understanding for the work packages to then make sense.

5) NPT is mentioned in WP2 but no rationale is provided for the adoption of this. Also NPT is abbreviated a few times first then defined in a later paragraph.

6) WP3 stage 2, clarify or provide example of the professional groups who will be approached, to ensure 'representation of diverse professional backgrounds'.

7) the authors acknowledge the limitation of only involving physicians in WP1 and 2, but what was the rationale for that?

8) Was there any PPIE involvement in the design of this study? Have healthcare professionals or stakeholders from the provider hospital been involved?

VERSION 1 - AUTHOR RESPONSE

Reviewer 1

**Comment**	**Authors' response**
This protocol outlines a longitudinal case study design comprising three work packages, using mixed methods to explore the implementation of an AI powered healthcare administration system in one private healthcare provider in Sweden. It is well-written and the focus on professional boundaries, AI literacy requirements, implementation challenges and impact on work conditions suggest this will be a very interesting, relevant and timely piece of research.	We thank the reviewer for their positive assessment of our manuscript and for the constructive feedback.
1) Setting - it isn't clear whether the sample is from one particular provider site or across several sites. The authors state that the provider (Capio) has 12,000 employees in Sweden. How are the sites to be selected?	1) We thank the reviewer for pointing this out. To clarify, we will recruit participants from care facilities at Capio that have implemented the AI system (12 primary care and 20 specialist care facilities). In the revised manuscript, we provide detailed information about the study setting (page 4):<br>"*The study setting will focus on 12 primary care facilities and 20 specialist care facilities at Capio in Sweden that have implemented the AI system*".

2) Sample - linked to this, how will the 20 physicians from PC and 20 physicians from specialist care be identified and approached?	2) We have added a detailed description of participant selection and recruitment under the subsection "Participant Recruitment and Sampling Strategy". Site leaders and secretaries will provide a list of physicians actively using the AI system at each selected site, stratified by care setting (primary vs. specialist). From this list, we will use purposive sampling to recruit healthcare professionals with different genders, work experience and diverse clinical specialties (for specialist care: representing different specialization areas such as internal medicine, psychiatry, and orthopedics), but with similar time of exposure to the AI-powered healthcare system. This approach ensures diversity in demographic and professional characteristics while controlling for implementation experience.<br>Potential participants will be informed about the research by the secretary and site leadership, and interview appointments will be scheduled by the study authors with secretarial support to ensure voluntary participation. Written information about the study (aims, time commitment, confidentiality, right to withdraw) will be provided to all potential participants. If participants withdraw, we will recruit replacement physicians using the same purposive sampling approach to maintain sample diversity and size.
3) The AI system - the detail provided is a bit vague. Is it a bespoke system created by/for Ramsay Sante or is it utilised elsewhere? Has there been any other pilot or evaluation of the system elsewhere?	3) We thank the reviewer for this observation. In response to your question, we have substantially rewritten the subsection "The AI System" (page 5) to address all aspects of this question. We now clarify that the AI system is not a bespoke system created exclusively for

	Capio Ramsay Santé, but a commercial certified platform developed by a Swedish health-tech company and currently being adopted across multiple healthcare organizations in Sweden and other Nordic countries. The platform was launched in 2023 and has been tested across multiple healthcare settings in Sweden.<br>The revised section provides detailed description of the system's technological components and functionality, including: the ambient scribe technology that uses Natural Language Processing and speech recognition to capture physician-patient conversations; machine learning algorithms for automated medical coding; large language models for generating clinical documentation; and integrated coordination platforms for multidisciplinary team communication. We also describe how the system operates across three phases of clinical workflow (preparation, consultation, and post-consultation).
4) I would move the sections above describing setting, sample and AI system to nearer the start of the methods, after the 'overall design' section. Readers need this context and understanding for the work packages to then make sense.	4) Following your recommendation, we have reorganized the "Methods and Analysis" section to improve logical flow. The new structure is:<br>• Study Design<br>• Study Setting<br>• The AI System<br>• Participant Recruitment and Sampling Strategy<br>• WP1: Exploring boundaries and literacy<br>• WP2: Exploring implementation challenges<br>• WP3: Exploring impact<br>• Patient and Public Involvement
5) NPT is mentioned in WP2 but no rationale is provided for the adoption of this. Also NPT is	5) We thank the reviewer for this observation. We have corrected the

abbreviated a few times first then defined in a later paragraph.	abbreviation usage throughout the manuscript. Normalization Process Theory (NPT) is now properly introduced with its full term before the abbreviation is used.<br>We have added explicit rationale for selecting NPT as the theoretical framework in the WP2 section. Under the subsection "WP2: Exploring implementation challenges", we now explain the justification for using NPT. At the end of the first paragraph, we state:<br>*"Normalization Process Theory (NPT) was selected as the analytical framework for this work package because it specifically addresses how complex interventions become or do not become part of everyday routines (embedded) and are transformed into sustainable practices [59]".*
6) WP3 stage 2, clarify or provide example of the professional groups who will be approached, to ensure 'representation of diverse professional backgrounds'.	6) We have added specific details in the WP3 Stage 2 description:<br>"*We will conduct focus groups with various professional groups (e.g., physicians, registered nurses, medical secretaries, healthcare managers) across both business areas to gather qualitative data about their experiences, perceptions, and perspectives regarding the value and effectiveness of the administration system*".
7) The authors acknowledge the limitation of only involving physicians in WP1 and 2, but what was the rationale for that?	7) The decision to focus exclusively on physicians in WP1 and WP2 is mainly based on two considerations:<br><br>**a) Professional boundary work dynamics:** WP1 specifically investigates professional boundary work, a concept primarily theorized around professions with high jurisdictional control and autonomy

	(Abbott, 1988). Physicians represent the archetypal case for studying professional boundary negotiations in healthcare due to their central role in clinical decision-making and their historical position of professional dominance (Coburn, 2006). While other healthcare workers certainly engage in boundary work, physicians' boundary work has distinct characteristics related to medical authority, diagnostic responsibility, and treatment decisions that require focused investigation. Physicians, as the most autonomous group in healthcare, provide a critical case for understanding how professionals respond when AI technologies challenge their established areas of authority (Lombi & Rossero, 2024).<br><br>**b) Primary AI system users:** The AI-powered system's core functionalities (ambient documentation, medical coding, clinical summarization) are primarily designed for physician workflows. While nurses, administrative staff, and other professionals may interact with the AI-generated outputs, physicians are the primary active users who directly engage with the system's AI capabilities during clinical work. This usage pattern makes physicians the most appropriate participant group for exploring AI literacy requirements (RO2) and implementation normalization processes (RO3).
8) Was there any PPIE involvement in the design of this study? Have healthcare professionals or stakeholders from the provider hospital been involved?	8) We have addressed this in the new "Patient and Public Involvement" section (added per Editor Comment 7).

References

Abbott, A. (1988). *The System of Professions. An essay on the division of expert labour.* London: The University of Chicago Press.

Coburn, D. (2006). Medical dominance then and now: Critical reflections. *Health Sociology Review*, *15*(5), 432-443. https://doi.org/10.5172/hesr.2006.15.5.432

Lombi, L., & Rossero, E. (2024). How artificial intelligence is reshaping the autonomy and boundary work of radiologists. A qualitative study. *Sociology of Health & Illness*, *46*(2), 200-218. https://doi.org/10.1111/1467-9566.13702

Merriam, S. B., & Tisdell, E. J. (2016). *Qualitative research in practice: A guide to design and implementation* (4 ed.). Wiley.

Swedish Research Council (2024). Good Research Practice 2024.

World Medical Association (2013). Declaration of Helsinki: Ethical Principles for Medical Research Involving Human Subjects. *JAMA, 310*(20), 2191-2194. https://doi.org/10.1001/jama.2013.281053

VERSION 2 - REVIEW

Reviewer 1

Name Bradley, Fay

Affiliation The University of Manchester, Division of Nursing, Midwifery & Social Work, School of Health Sciences

Date 05-Mar-2026

COI

Thank you for the opportunity to review this revised version. I confirm that I am satisfied that the authors have addressed my comments and I recommend the protocol for publication.
